# Efficacy of a Combined Treatment of Botulinum Toxin and Intensive Physiotherapy in Hereditary Spastic Paraplegia

**DOI:** 10.3389/fnins.2020.00111

**Published:** 2020-02-21

**Authors:** Gabriella Paparella, Marinela Vavla, Lisa Bernardi, Giulia Girardi, Cristina Stefan, Andrea Martinuzzi

**Affiliations:** ^1^Acquired Neuropsychological Disease Rehabilitation Unit, Scientific Institute, IRCCS Eugenio Medea, Pieve di Soligo, Italy; ^2^Severe Developmental Disabilities Unit, Scientific Institute, IRCCS Eugenio Medea, Conegliano, Italy

**Keywords:** hereditary spastic paraplegia, spasticity, lower limbs, botulinum toxin A, physiotherapy

## Abstract

**Introduction:**

The Hereditary Spastic Paraplegia (HSP) is a heterogeneous group of neurodegenerative disorders characterized by progressive spasticity and lower limbs (LL) weakness. There is no treatment to cure or halt the disease, except for symptomatic therapy. The use of botulinum toxin type A (BoNT-A) is one of the primary treatment for focal spasticity. Physiotherapy (PT) can help in maintaining residual functioning. We performed a retrospective study to evaluate the effect of the combined BoNT-A and intensive PT in patients with HSP.

**Methods:**

Eighteen adult patients (50% females) with clinical diagnosis of HSP were recruited. Eleven patients had a genetic diagnosis of SPG4, 5, 7, 8, 11, 72. Patients were all autonomously deambulant or needed support. BoNT-A was injected in 36 LL in different spastic muscles under electromyographic guidance and followed by intensive PT sessions. Outcome measures included disease severity, motor functional measures, perceived pain self-report and quality of life. Assessments occurred at baseline, 1 and 3 months after BoNT-A injection.

**Results:**

Most inoculated muscles were hamstrings, rectus femoris and gastrocnemius. We observed an improvement in muscle tone, in the gait velocity and distance length. Spastic Paraplegia Rating Scale was significantly reduced after treatment, in addition to improving pain and quality of life. These results were riconfirmed in 3 months time.

**Conclusion:**

Our study indicates that combined treatment of BoNT-A and PT can lead to improvement of spasticity and quality of life in patients with HSP.

## Introduction

The Hereditary Spastic Paraplegia (HSP) or Strumpell-Lorrain syndrome comprises a heterogeneous group of neurodegenerative disorders caused by axonal retrograde degeneration of the long corticospinal tracts (Fink, 2014; Lo Giudice et al., 2014; Klebe et al., 2015; de Souza et al., 2017). HSP presents with progressive spasticity and weakness of the lower limbs (LL), reduced vibration sense, hyperactive deep tendon reflexes and inconstant urinary urgency (Fink, 2014; Lo Giudice et al., 2014; Klebe et al., 2015; de Souza et al., 2017).

The HSP prevalence is difficult to estimate due to different data present in literature. According to a recent epidemiological survey, the world-wide prevalence of HSP is 1.8 – 5.5: 10^5^, with variability depending on the geographical area considered (Ruano et al., 2014). The age of onset is variable, ranging from early childhood to the eighth decade (Fink, 2014; Lo Giudice et al., 2014; Klebe et al., 2015). Currently, more than 85 genomic loci and 79 mutated genes associated with HSP have been identified, highlighting the extreme heterogeneity in the mode of transmission and in the role of encoded proteins (Parodi et al., 2018). The prognosis and the severity of the clinical picture in HSP varies between families and, to a lesser extent, within the same family, although life expectancy is normal. When the disease occurs in early childhood, the symptoms may not progress significantly for several years and decades; on the contrary, the late-onset HSPs might be associated with a more insidious worsening pattern (Harding, 1993). Finally, it has been reported that, while in the pure forms patients rarely resort to using the wheelchair but frequently walk with aids, in the complex forms the functional disability depends on the extent and severity of extra-motor involvement (Lo Giudice et al., 2014).

The LL spasticity, often accompanied by muscle weakness, is the key clinical sign of HSP that affects a wide group of muscles such as the hamstrings, quadriceps, adductors, gastrocnemius and soleus. In particular, the weakness primarily concerns the iliopsoas, hamstring muscles, and tibialis anterior (Fink, 2014). Typically, there is a strong discrepancy between the severity of spasticity, often present at disease onset, and the weakness which might be subtler and appear at a late stage of disease (McDermott et al., 2000). There is no specific treatment for HSP to prevent or reduce the gait impairment and disability progression. Current therapies are symptomatic and aim to prevent complications such as multiple LL muscle contractures, pain and fractures, and also aim to improve patients’ quality of life (de Souza et al., 2017).

Physiotherapy (PT) is generally recommended to improve articulation, maintain and increase LL strength, prevent muscle atrophy, delay or prevent contractures or deformities, improve cardiovascular endurance and mobility in spastic patients (Fink, 2013). Even though frequently prescribed and performed, little experience has been reported in regard to the PT in HSP patients.

The selective chemodenervation via intramuscular injection of botulinum toxin type A (BoNT-A) is widely used in the treatment of focal spasticity in patients with stroke, multiple sclerosis, spinal cord injuries and various neurological disorders (Dunne et al., 1995; Keren et al., 2000; Ward, 2008). There have been several reports on the use of botulinum toxin in HSP patients but with discrepancy of the various clinical protocols (Rousseaux et al., 2007; Hecht et al., 2008; Geva-Dayan et al., 2010; de Niet et al., 2015; Riccardo et al., 2016; Servelhere et al., 2018). Previous studies demonstrate the effectiveness of toxin injection at the level of triceps sural and/or of the adductors muscles as beneficial in reducing the spasticity and increasing the walking speed (Rousseaux et al., 2007; Hecht et al., 2008; de Niet et al., 2015). However, functional improvements have been found only in a limited number of patients with a clinical diagnosis of HSP. The motor and non-motor effects of BoNT-A were described in 33 patients with definite HSP diagnosis (Servelhere et al., 2018), reporting improvement in terms of adductors tone reduction and perceived fatigue. The muscles more frequently inoculated were: triceps sural and adductors. The muscles more inoculated were: triceps sural and adductors. Toxin infiltration was performed with only palpation as a guide and this could limit the accuracy and effectiveness of the treatment. Most of the other studies (Hecht et al., 2008; de Niet et al., 2015; Riccardo et al., 2016) evaluated the functional effects of botulinum toxin associated with PT treatment in patients with HSP. However, none of these studies disclosed the PT rehabilitation program to which patients were subjected.

The objective of this study was to investigate the effects of a combined treatment of BoNT-A injection and subsequent intensive PT in the management of spasticity in adult HSP patients. This experience could provide an indication for a rehabilitation protocol in these patients.

## Materials and Methods

### Design

**FIGURE 1:**
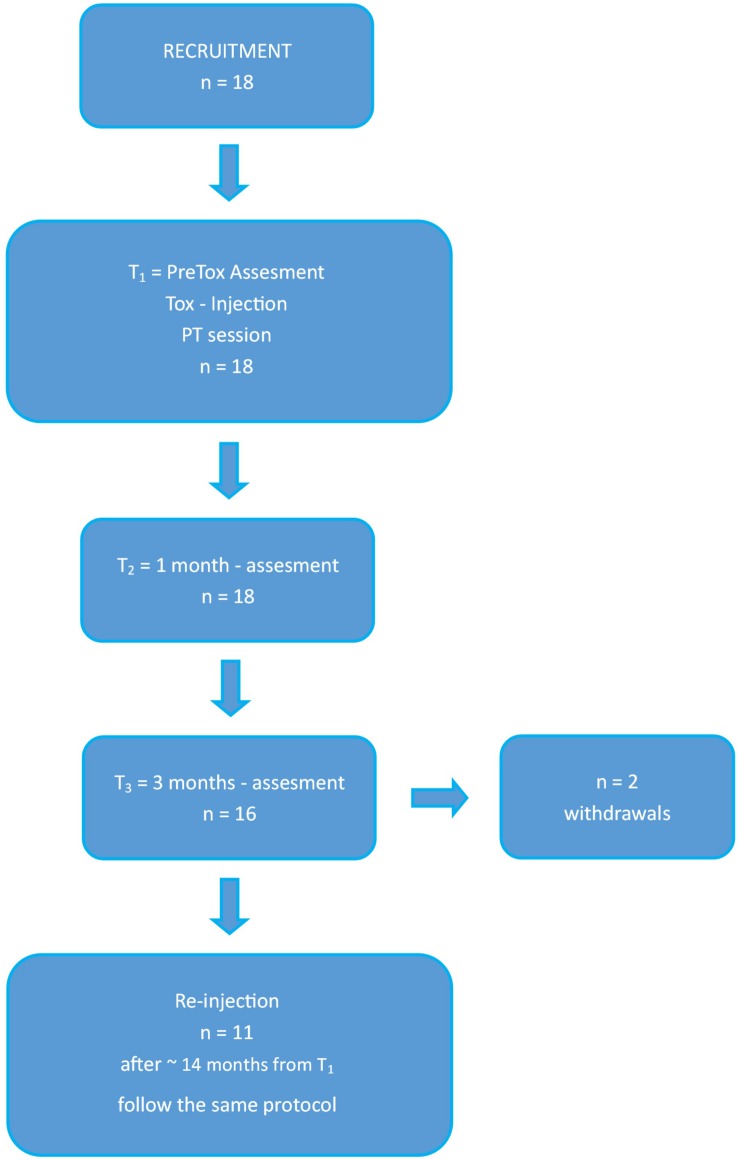
Study scheme. Working flowchart used for this study. Tox, toxin, PT, physiotherapy, T1, assessment at baseline; T2, assessment at 1 month; T3, assessment at 3 months.

This was a retrospective observational cohort study, as classified by the Institutional Review Board (IRB). The study scheme is represented in Figure 1.

### Participants

A total of 18 patients with clinical diagnosis of HSP treated with BoNT-A injections were recruited at the “Eugenio Medea” Scientific Institute in Pieve di Soligo (Treviso, Italy) from 2013 until 2018. Our study has been approved by the Institutional Ethics Committee of “Eugenio Medea” Research Institute (# 63/09CE) and was conducted in accordance to the ethical standards of the Declaration of Helsinki (World Medical Association, 1964). All participants were informed about the experimental nature of the study and signed an informed consent for participation. During treatment patients had not been subjected to any other form of PT in addition to the program scheduled in the study protocol. The inclusion criteria were: clinical diagnosis of HSP, confirmed or ongoing genetic investigation, aged 18 years and older, ambulant patients with or without walking aid. Moreover, the recruited patients had been referred to our institute for LL BoNT-A injection. Exclusion criteria included any orthopedic injury that could eventually limit their ability to mobilize and/or any clinical condition that could affect their motor skills.

### Intervention

#### BoNT-A Injection

All patients received intramuscular injection of BoNT-A under electromyographic guidance. Three forms of BoNT-A were used: Xeomin (Merz), Dysport (Ipsen) and Botox (Allergan). The dose of botulinum toxin was determined after careful evaluation of each patient’s weight, degree of spasticity, number and type of target muscles injected. The injections were repeated twice with a minimum inter-injection interval of 3 months in 11 of the 18 enrolled patients.

#### PT Protocol

After the focal chemodenervation, the patients underwent intensive PT, according to their functionality, impairments and goals. The intervention was an individualized inpatient rehabilitation program consisting in 2 h PT daily for overall 10 sessions. The focus of the PT program was to improve physical abilities, in particular postural control, balance abilities and gait pattern that would subsequently prevent falls, inactivity, reduce the fatigue in walking in order to maintain the independence.

The PT protocol followed some key points given below:
(1) Stretching and mobilizing in order to lengthen the inoculated muscles;
(2) Postural control and trunk stability with activation of deep trunk musculature and paravertebral musculature;
(3) Foot core training;
(4) Stimulation of anticipatory postural adjustments in order to reduce compensations or fixation, following an appropriate orientation of LL;
(5) Strengthening focused on LL (in particular of antagonist muscles) and trunk muscles. The muscle strengthening should be specific, without the use of rewards on motor control. The weak muscles are usually represented from hip extensor and abductors, knee extensor and ankle dorsal flexors, that are important even in the antigravity postural control;
(6) Gait training to improve gait pattern and reduce the risk of falls;
(7) Cardiovascular activity that includes cycle ergometer, treadmill and gait trainer. This improves muscular endurance and strength, helps integrating learned patterns and prevents the weakness of not activated muscles.

#### Outcome Measures

Assessments occurred at baseline (T1), one (T2), and 3 months (T3) after BoNT-A injection. Patients were assessed according to a specific clinical protocol executed by the same physiotherapist.

The assessment consisted in the following measures:
– Spastic Paraplegia Rating Scale (SPRS, Schüle et al., 2006), which has been approved as a valid measure of disease severity in HSP. It has a maximum score of 52 (maximum severity) and a minimum of 0 (normal).
– Walking Handicap Scale (WHS, Perry et al., 1995) which evaluates the level of functional walking ability at home and in the community. This scale ranges from 1 (physiological walker) to 6 (community walker).
– The Modified Ashworth Scale (MAS, Bohannon and Smith, 1987) used to measure the spasticity. The MAS measures resistance during the passive stretch of a joint. This scale ranges from 0 (normal tone) to 4 (rigidity of the joint).
– The 10 Meter Walking Test (10MWT, Rossier and Wade, 2001) and 2 Minute Walking Test (2MWT, Bohannon et al., 2015) used to measure the deambulation skills. These scales measure velocity and resistance of gait. We consider only comfortable gait as a normal speed gait.
– Timed UP and Go test (TUG, Podsiadlo and Richardson, 1991) assesses functional mobility, walking ability, balance and risk of falls.
– The Visual Analogical Scale (VAS, Hayes and Patterson, 1921) and Numeric Rating Scale (NRS, Williamson and Hoggart, 2005) which assess the perceived quality of life and pain respectively. Both scales range from 0 (no pain/none satisfaction in quality of life) to 10 (severe pain/full satisfaction in quality of life).

#### Statistical Analysis

Linear mixed models were used to study the relationship between the results of each test (i.e., 10MWT, 2MWT, TUG, WHS, VAS, NRS, SPRS) at the three time points (fixed effect), considering the subject as a random effect. The “lme4” R package was used to fit the aforementioned models (Bates et al., 2015). *Post hoc* tests were used to pairwise compare the values between time points (i.e., T1 vs. T2, T1 vs. T3, and T2 vs. T3), based on *t*-Student distribution with degrees of freedom (df) computed by the Kenward-Rogers method using the “emmeans” R package (Lenth, 2019).

Similarly, in order to study the MAS values that were measured on an ordinal scale, we adopted a cumulative link mixed model that handles the ordered nature of the data. The time points (T1, T2, and T3) and the LL (right or left) were considered as fixed effects, while the subject was incorporated in each model as random effect. The “ordinal” R package was used to fit these models (Christensen, 2019). *Post hoc* tests were used to pairwise compare the MAS values between time points (i.e., T1 vs. T2, T1 vs. T3, and T2 vs. T3), based on asymptotic approximation using the “emmeans” R package (Lenth, 2019). Results of the *post hoc* tests were computed considering the average over the right and left MAS values of each muscle. Correction of the *post hoc* tests for multiple comparisons was performed with Benjamini-Hochberg (false discovery rate) procedure. Adjusted *P* < 0.05 was considered significant. Analyses were done in R (version 3.6.0).

## Results

### Patients

According to defined inclusion criteria we recruited 18 patients with mean age of 53.9 ± 12.2 years, disease duration of 9.5 ± 5.3 years and age at onset of 44.4 ± 13.01 years. There were nine females. All patients included in the study were able to walk with (*n* = 6) or without (*n* = 12) walking aids on a level surface. Defined molecular diagnosis of HSP was reached in eleven patients: SPG4 was the most frequent genotype (27.8%); the remaining study population had SPG8, 5, 7, 11, 72 diagnosis. Seven patients did not have a defined molecular diagnosis at the time of examination, but they showed either a recessive (*n* = 3) or dominant (*n* = 4) inheritance pattern. The demographic and clinical data of these subjects are presented in Table 1.

**TABLE 1:** Demographic and clinical data of patients included in this study.

**Variable**	**Patients *n* = 18**
Gender *M/F*	9/9

	**Mean ± SD**
	**(Range)**

Age, years	53.9 ± 12.2
	(30.7–75.2)
Age at onset, years	44.4 ± 13.0
	(26–70)
Disease duration, years	9.5 ± 5.3
	(2.5–22.2)

*Genotype*	***n (%)***

SPG4	5 (27.8)
SPG5	1 (5.55)
SPG7	1 (5.55)
SPG8	2 (11.1)
SPG11	1 (5.55)
SPG72	1 (5.55)
Molecular diagnosis ongoing	7 (38.9)

*Deambulation*	***n (%)***

Independent	12 (66.7)
With a cane	3 (16.6)
With a walker	2 (11.1)
With crutches	1 (5.6)

*M, male; F, female; SD, standard deviation; SPG, spastic paraplegia gene.*

#### Intervention and Evaluations

All patients included in the study were given intramuscular injections of BoNT-A and afterward underwent the intensive PT sessions, completing the proposed treatment. BoNT-A was injected in 36 LL (18 right LL, 18 left LL), for a total of 85 muscles. Most inoculated muscles were hamstrings (*n* = 28), rectus femoris (*n* = 28), gastrocnemius (*n* = 13) and adductors (*n* = 8). None of the patients enrolled had side effects after treatment. All patients have been evaluated according to the clinical protocol. Only two patients have not been assessed 3 months after BoNT-A injection (T3) for missed appointments.

#### Variations of Modified Ashworth Scale in Time

**FIGURE 2:**
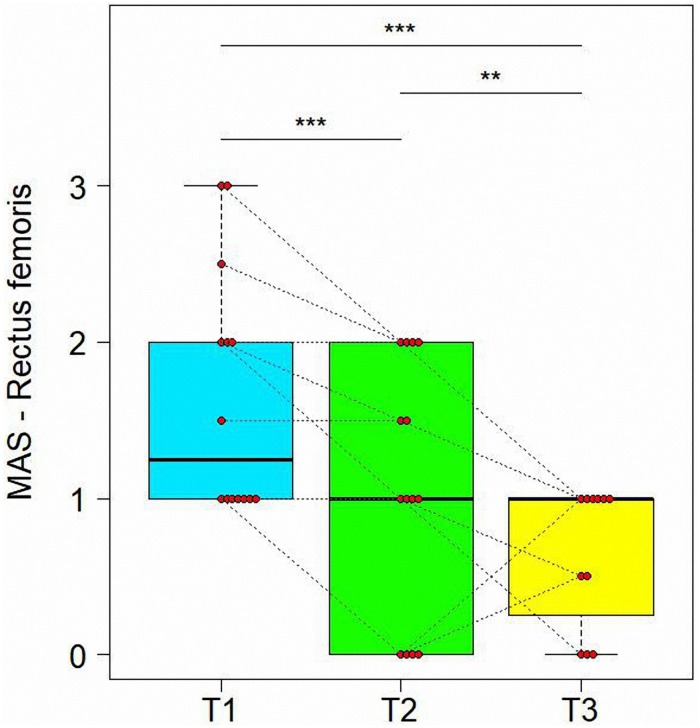
Variations of Modified Ashworth scores (MAS) in time for Rectus Femoris. Data are represented by boxplots at three time point: before the treatment (T1), after 1 month (T2) and after 3 months (T3) from the treatment. At each time, individual values of patients are also shown as red points. Values of the same subject at different times are connected by a line. Significant differences between times are represented with asterisks: **0.001 < *P* < 0.01, ***P < 0.001.

**FIGURE 3:**
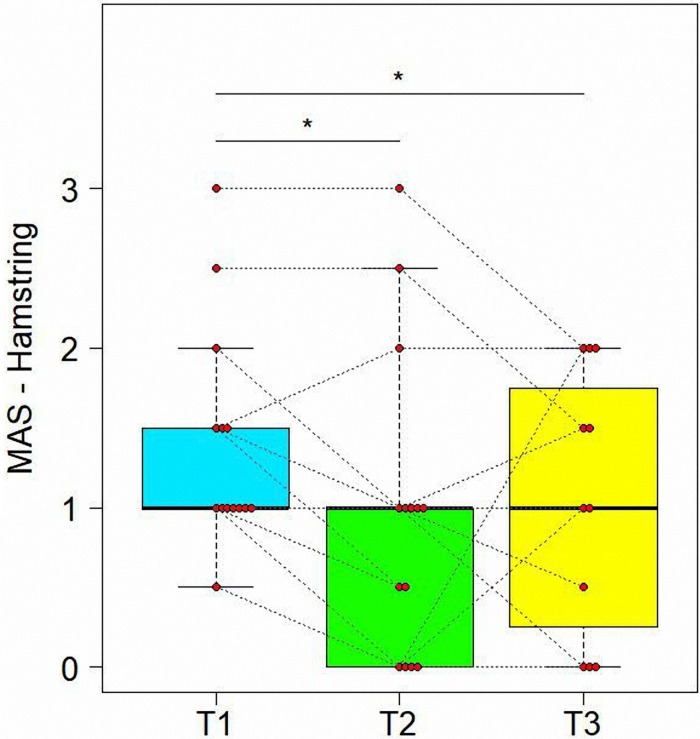
Variations of Modified Ashworth scores (MAS) in time for Hamstring. Data are represented by boxplots at three time points: before the treatment (T1), after 1 month (T2) and after 3 months (T3) from the treatment. At each time, individual values of patients are also shown as red points. Values of the same subject at different times are connected by a line. Significant differences between times are represented with asterisks: *0.01 < *P* < 0.05.

Table 2 presents variation of spasticity in time for the muscles injected. As shown in Table 3, MAS values were significantly reduced from T1 to T2 for rectus femoris (z = −4.086, adjusted *P* = 0.0004) and hamstring (*z* = −2.787, adjusted *P* = 0.0166) with rectus femoris MAS values decreasing further from T2 and T3 (*z* = −3.242, adjusted *P* = 0.0071) (see Figures 2, 3). Finally, treatment also resulted in a significant reduction in gastrocnemius spasticity from T1 to T2 (*z* = −112.825, adjusted *P* = 0.0166).

**TABLE 2:** Evolution of Modified Ashworth scores in time.

**Muscle**	**N**	**Mean ± SD (Min-Max)**		
		**T1**	**T2**	**T3**
Rectus femoris	28	1.6 ± 0.7 (1–3)	1.1 ± 0.8 (0–2)	0.6 ± 0.5 (0–2)
Hamstring	28	1.3 ± 0.7 (0–3)	0.9 ± 0.9 (0–3)	1 ± 0.8 (0–2)
Adductors	8	2 ± 0.5 (1–3)	1.4 ± 0.9 (0–3)	0.9 ± 0.6 (0–2)
Gastrocnemius	13	1.7 ± 0.9 (1–3)	0.8 ± 0.8 (0–3)	1.33 ± 1.1 (0–3)
Soleus	5	1.8 ± 1 (1–3)	1 ± 1.4 (0–3)	1 ± 1.2 (0–3)

*T1, assessment at baseline; T2, assessment at 1 month; T3, assessment at 3 months.*

**TABLE 3:** Results of the *post hoc* comparisons of the Modified Ashworth scores between time points.

**Muscle**	**First injection**		
	**T1 vs. T2**	**T1 vs. T3**	**T2 vs. T3**
Rectus Femoris	**0.0004**	**<0.0001**	**0.0071**
Hamstring	**0.0166**	**0.0447**	0.9058
Adductors	0.0561	**0.0166**	0.0956
Gastrocnemius	**0.0166**	0.0818	0.1211
Soleus	0.1631	0.1916	0.8556

*Data are adjusted *P*-values with Benjamini-Hochberg correction. Numbers in bold: adjusted *P* < 0.05. T1, assessment at baseline; T2, assessment at 1 month; T3, assessment at 3 months.*

#### Variations of Functional Scales in Time

**FIGURE 4:**
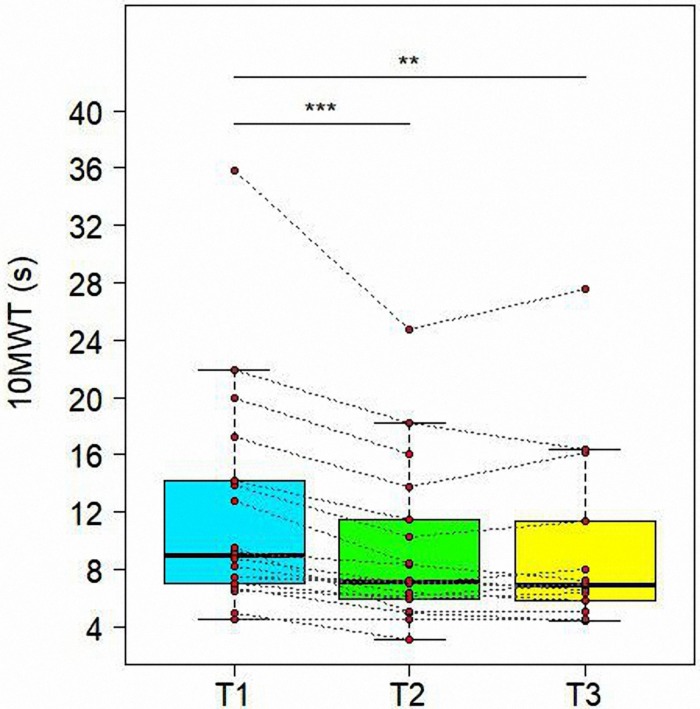
Variations of 10 Meter Walk Test (10MWT) in time. Data are represented by boxplots at three time points: before the treatment (T1), after 1 month (T2) and after 3 months (T3) from the treatment. At each time, individual values of patients are also shown as red points. Values of the same subject at different times are connected by a line. Significant differences between times are represented with asterisks: **0.001 < *P* < 0.01, ***P < 0.001.

Table 4 presents evolutions of other evaluation scales in time. First of all, treatment resulted in a significant improvement in the 10MWT (see Figure 4). We observed a significant variation from T1 to T3 for the values of all scales, specifically a reduction for 10MWT (*t* = −4.034, *df* = 30, adjusted *P* = 0.0012), TUG (*t* = −2.477, *df* = 30, adjusted *P* = 0.0364), and an increase for 2MWT (*t* = 4.116, *df* = 30, adjusted *P* = 0.0012) and WHS (*t* = 3.185, *df* = 27, adjusted *P* = 0.0084) (Table 5). Similar significant variations were found also between T1 and T2 for 10MWT (*t* = −5.136, *df* = 30, adjusted *P* = 0.0002) and 2MWT (*t* = 3.277, *df* = 30, adjusted *P* = 0.0080).

**TABLE 4:** Evolution of other evaluation scales in time.

**Scale**	**T1**			**T2**			**T3**		
	**n**	**Median**	**Range (min-max)**	**n**	**Median**	**Range (min-max)**	**n**	**Median**	**Range (min-max)**
10MWT *(s)*	18	9	4.5–35.8	18	7.1	3.1–24.7	14	7	4.4–27.6
2MWT *(m)*	18	78.5	22–179	18	99.5	29.5–184	14	99.5	27.5–190.7
TUG *(s)*	18	14.3	6.5–55	18	12.5	6.5–51	14	11.9	5.2–49.5
WHS *(1–6)*	16	5	2–5	16	5	2–6	13	5	3–6
VAS *(0–10)*	12	4	2–8	12	4	0–8	10	4	0–6
NRS *(0–10)*	18	5	1–8	18	3.5	0–6	14	3	0–6
SPRS *(0–52)*	18	17	7–36	18	17	6–35	14	15.5	6–34

*10MWT, 10 Meter Walk Test; 2MWT, 2 Minute Walk Test; TUG, Timed Up and Go; WHS, Walking Handicap Scale; VAS, Visual Analogical Scale; NRS, Numeric Rating Scale; SPRS, Spastic Paraplegia Rating Scale; T1, assessment at baseline; T2, assessment at 1 month; T3, assessment at 3 months.*

**TABLE 5:** Results of the *post hoc* comparisons of the evaluation scales between time points.

**Scale**	**First injection**		
	**T1 vs. T2**	**T1 vs. T3**	**T2 vs. T3**
10MWT	**0.0002**	**0.0012**	0.5335
2MWT	**0.0080**	**0.0012**	0.2907
TUG	0.1408	**0.0364**	0.3731
WHS	0.1408	**0.0084**	0.1616
VAS	0.2472	**0.0084**	0.0949
NRS	**0.0012**	**0.0002**	0.1616
SPRS	**0.0111**	**0.0004**	0.0984

*Data are adjusted *P*-values with Benjamini-Hochberg correction. Numbers in bold: adjusted *P* < 0.05. 10MWT, 10 Meter Walk Test; 2MWT, 2 Minute Walk Test; TUG, Timed Up and Go; WHS, Walking Handicap Scale; VAS, Visual Analogical Scale; NRS, Numeric Rating Scale; SPRS, Spastic Paraplegia Rating Scale; T1, assessment at baseline; T2, assessment at 1 month; T3, assessment at 3 months.*

#### Variations of VAS and NRS Scores in Time

**FIGURE 5:**
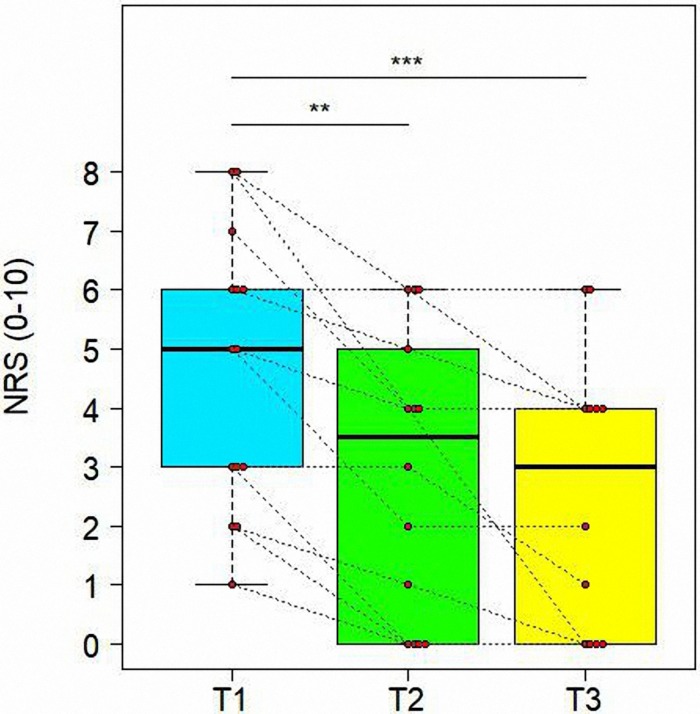
Variations of Numeric Rating Scale (NRS) in time. Data are represented by boxplots at three time points: before the treatment (T1), after 1 month (T2) and after 3 months (T3) from the treatment. At each time, individual values of patients are also shown as red points. Values of the same subject at different times are connected by a line. Significant differences between times are represented with asterisks: **0.001 < *P* < 0.01, ***P < 0.001.

As reported in Table 5, there was a significant reduction from T1 to T3 for VAS (*t* = 3.36, *df* = 19, adjusted *P* = 0.0084) and NRS (*t* = −5.500, *df* = 24, adjusted *P* = 0.0002) scores. We also found a significant improvement from T1 to T2 for NRS (*t* = −4.170, *df* = 24, adjusted *P* = 0.0012) (see Figure 5).

#### Variations of SPRS Scores in Time

**FIGURE 6:**
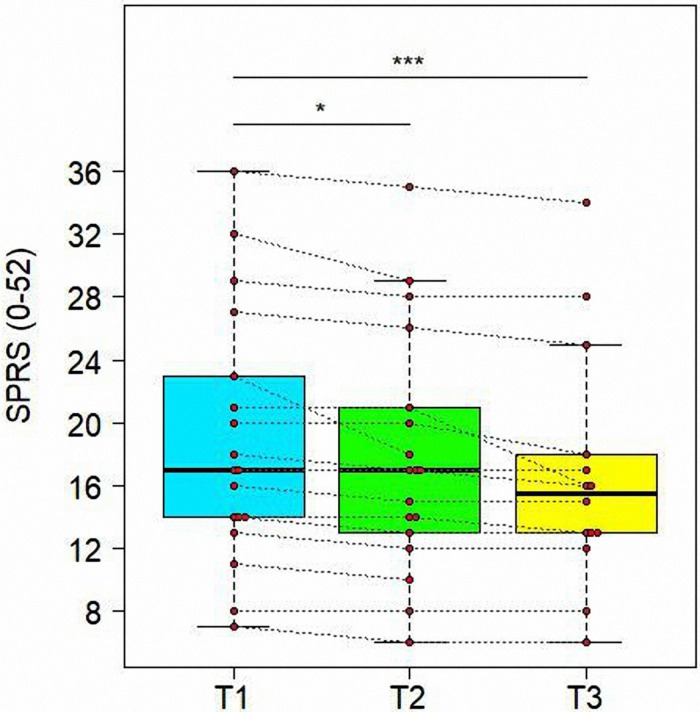
Variations of Spastic Paraplegia Rating Scale (SPRS) in time. Data are represented by boxplots at three time points: before the treatment (T1), after 1 month (T2) and after 3 months (T3) from the treatment. At each time, individual values of patients are also shown as red points. Values of the same subject at different times are connected by a line. Significant differences between times are represented with asterisks: *0.01 < *P* < 0.05, ***P < 0.001.

The main result of BoTN-A injections and intensive PT was a reduction of disease severity, highlighted by evolution of SPRS in time (see Figure 6). In fact, we demonstrate a significant reduction from T1 to T3 for SPRS score (*t* = −4.677, *df* = 30, adjusted *P* = 0.0004). Similar significant improvement was found also between T1 and T2 (*t* = −3.008, *df* = 30, adjusted *P* = 0.0111), as shown in Table 5.

### Re-injection

Of the initial group (*n* = 18), eleven patients underwent a second combined treatment of BoTN-A and intensive PT, after 1.2 ± 0.4 years from the first one. As the first injection, most injected muscles were rectus femoris (*n* = 16), hamstring (*n* = 12), and gastrocnemius (*n* = 8). All these patients were subjected a second standardized clinical evaluation, which highlighted results comparable to those obtained at first injection. In particular, as shown in Table 6, the same muscles showed lower MAS values at T2 than T1, but they were not significantly different between T2 and T3. Analogous results were also observed for NRS scale, that had significantly lower values only at T3 with respect to both T1 (*t* = −3.836, *df* = 16, adjusted *P* = 0.0075) and T2 (*t* = −2.542, *df* = 16, adjusted *P* = 0.0450). By contrast, TUG values were no longer statistically different between the three time points, as shown in Table 7.

**TABLE 6:** Results of the *post hoc* comparisons of the Modified Ashworth scores between time points at the second injection.

**Muscle**	**Second injection**		
	**T1 vs. T2**	**T1 vs. T3**	**T2 vs. T3**
Rectus Femoris	**0.0131**	**0.0131**	0.9885
Hamstring	**0.0303**	**0.0131**	0.3094
Adductors	NA	NA	NA
Gastrocnemius	**0.0303**	**0.0271**	0.0748
Soleus	NA	NA	NA

*Data are adjusted *P*-values with Benjamini-Hochberg correction. Numbers in bold: adjusted *P* < 0.05. NA = Not Available due to limited sample size. T1, assessment at baseline; T2, assessment at 1 month; T3, assessment at 3 months.*

**TABLE 7:** Results of the *post hoc* comparisons of the other evaluation scales between time points at second injection.

**Scale**	**Second injection**		
	**T1 vs. T2**	**T1 vs. T3**	**T2 vs. T3**
10MWT	**0.0241**	**0.0258**	0.9673
2MWT	**0.0248**	0.2841	0.2890
TUG	0.0682	0.2841	0.4935
WHS	0.2890	**0.0159**	0.1000
VAS	**0.0071**	**0.0035**	0.4935
NRS	0.3094	**0.0075**	0.0450
SPRS	**0.0130**	**0.0035**	0.4062

*Data are adjusted *P*-values with Benjamini-Hochberg correction. Numbers in bold: adjusted *P* < 0.05. 10MWT, 10 Meter Walk Test; 2MWT, 2 Minute Walk Test; TUG, Timed Up and Go; WHS, Walking Handicap Scale; VAS, Visual Analogical Scale; NRS, Numeric Rating Scale; SPRS, Spastic Paraplegia Rating Scale; T1, assessment at baseline; T2, assessment at 1 month; T3, assessment at 3 months.*

## Discussion

The aim of this study was to evaluate the effects of a combined treatment of BoTN-A followed by intensive PT in HSP patients with preserved walking ability. Our study showed significant functional improvement in all the outcome measures used, which was maintained for at least 3 months.

Although previous studies concerning BoTN-A treatment in HSP patients focused on distal musculature (gastrocnemius and soleus) or adductors, we decided to perform the BoTN-A treatment especially on hamstrings, rectus femoris, and, to a lesser extent, on gastrocnemius and adductors. Our choice was based on studies carried out in HSP patients with gait analysis: the spasticity of rectus femoris is often cause of an excessive antiversion of the pelvis on the sagittal plane and an excessive flexion of the hip during gait cycle (Piccinini et al., 2011). The Gait analysis highlighted hamstrings and rectus femoris co-contraction. This can reduce gait stability and patients may adopt expensive compensatory strategies at knee (e.g., hyperextension) and trunk (e.g., forward lean). Our study identified a short and medium-term improvement in rectus femoris and hamstrings spasticity. In accordance to previous studies, the reduction of the MAS was relatively modest. Nevertheless, HSP patients exhibit dynamic hypertonus during gait and perhaps it would be more appropriate to evaluate muscle spasticity with Gait Analysis.

From a functional point of view, the treatment proposed in this study gave rise to a significant increase in the comfortable gait velocity as evaluated with the 10MWT. These results are in agreement with several previous studies that considered the same outcome measure for evaluating the efficacy of the BoNT-A (Rousseaux et al., 2007; de Niet et al., 2015). Secondly, the combination of BoNT-A and the intensive PT has led to an increase of the distance walked in 2MWT and in exercise resistance. Indeed, the injection of some specific muscles (rectus femoris, hamstring and gastrocnemius), leads to a reduction in spasticity and less compensatory strategies implemented by patients during the walk, improvement of biomechanics and reduced energy consumption.

Thirdly, a significant improvement was obtained with the TUG test, in particular at 3 months after the BoNT-A injection. This result highlights the effectiveness of the combined treatment of BoNT-A and intensive PT in terms of better stability and increased capacity for selective muscle control. de Niet et al. (2015) previously administered this outcome measure, but in contrast they registered no post-treatment improvement. This discrepancy of results could be attributed to the difference in muscles treated (only plantar flexors) and also could be due to the differences in the PT program that consisted in independently performed single stretching exercise (de Niet et al., 2015). It is plausible that the lack of a specific PT program may have reduced the selective control capacity of patients.

Finally, a further functional improvement, was recorded in the WHS scale. In most cases, there has been a short-term improvement in the quality of the walking in the social sphere. This scale is less sensitive to variations because it is a coarse scale that includes only 6 functional categories. The treatment proposed in this study was demonstrated to be effective in terms of quality of life, with a significant improvement at 3 months. Our study also showed a significant improvement of the pain perceived by patients. Only one previous study has investigated the effectiveness of BoNT-A in HSP in terms of pain reduction, but showed no significant improvements (Servelhere et al., 2018). However, the same study did not combine the chemodenervation with PT and this may have mitigated the effects of the treatment. In our study, pain and the quality of life perceived by patients, improved significantly 3 months after treatment. We hypothesize that the intensive.

PT we proposed after the injection has conducted to a gradual training of the muscle chains and a progressive modification of the motor pattern. This has determined a reduction of pain and stiffness perceived either in static or dynamic posture in the medium-long term.

One of the major contributions of our study concerns the use of SPRS as an outcome measure of disease severity in HSP. The significant improvement on SPRS scores recorded both at 1 month, and especially at 3 months, highlights the effectiveness of the proposed intervention in terms of modifying disease severity. This could be also an indicator of an improved quality of life.

Another fundamental contribution of this study concerns the design of a combined protocol of BoNT-A injection with a specific PT programme that contributes to improvement of postural control, balance abilities and gait pattern. Use of BoNT-A combined with PT shows interesting results after the second chemodenervation, suggesting this formula as an appropriate therapy for an incurable disease. Finally, the intervention proposed in our study showed an excellent tolerability for all the patients since no adverse events were reported.

A limitation of this study is the small patient sample, as well the genetic and clinical heterogeneity of our cohort. In addition, there was no specific BoNT-A injection protocol, since the target muscles, botulinum type and administration of doses were left to the evaluating physicians. It was also a retrospective study with no control group.

Therefore, it is essential that future studies include larger cohorts of participants and a randomized controlled design. The combined treatment protocol of BoNT-A injection and PT could be compared to control groups such as intensive PT or BoNT-A only. In addition, it could be of interest to investigate the different treatment outcomes and protocols in various SPG types and distinct clinical phenotypes.

Future studies could systematically implement the use of more detailed techniques such as gait analysis that provides biomechanical measurement of the gait cycle. Although the MAS is the most widely used tool for assessing muscle tone, it is not very sensitive. For that reason, spasticity could be assessed more accurately also by the Tardieu Scale due to its better sensibility in differentiating the contractures from spasticity (Patrick and Ada, 2006). Moreover, the inclusion of an objective outcome measure such as the energy cost of walking, measured by cycle ergometry, could add strength to the results and provide a more rigorous rationale to explain the functional perceived improvement.

The results of this study highlight that a combined treatment of BoNT-A and intensive PT improves function for people with HSP. Larger randomized controlled trials are needed to confirm the results obtained with this retrospective study and to possibly validate the effectiveness of this approach. These results could provide meaningful treatment options for the care of HSP patients.

## Data Availability Statement

All datasets generated for this study are available on request.

## Ethics Statement

The studies involving human participants were reviewed and approved by the Institutional Ethics Committee of “Eugenio Medea” Research Institute (# 63/09CE) and were conducted in accordance to the ethical standards of the Declaration of Helsinki (World Medical Association, 1964). The patients/participants provided their written informed consent to participate in this study.

## Author Contributions

GP and LB collected and analyzed the data, and prepared the manuscript. GP, GG, and CS evaluated the patients and provided the data. GP and MV provided the design of the study. AM provided the design of the study, supervision and manuscript revision. All authors revised the manuscript for important intellectual content, provided approval for publication, and agreed to be accountable for all aspects of the work.

## Conflict of Interest

The authors declare that the research was conducted in the absence of any commercial or financial relationships that could be construed as a potential conflict of interest.
